# Genomic Signatures of Endemic Pathogen Exposure in a Declining Amphibian

**DOI:** 10.1111/mec.70603

**Published:** 2026-10-25

**Authors:** Alexander S. Wendt, Lee Berger, Lee F. Skerratt, Laura A. Brannelly

**Affiliations:** ^1^ Melbourne Veterinary School, Faculty of Science The University of Melbourne Werribee Victoria Australia

**Keywords:** adaptive variation, amphibian genomics, *Batrachochytrium dendrobatidis*, host‐pathogen interactions, pathogen‐mediated selection, polygenic adaptation

## Abstract

Endemic wildlife diseases can impose strong selection on threatened populations, yet whether persistence is associated with adaptive genomic responses remains poorly understood. We tested whether long‐term exposure to *Batrachochytrium dendrobatidis* (*Bd*) following population declines has left detectable selection signatures in the genome of the alpine tree frog, *Litoria verreauxii alpina*. Because prior work suggests limited immunity to *Bd* within the species, we did not expect persistence to be explained primarily by repeated shifts at canonical immune genes. Instead, we expected pathogen‐associated differentiation, if present, to be diffuse and weakly shared across contrasts, potentially involving regulatory, signalling or reproduction pathways consistent with tolerance, compensatory recruitment or demographic turnover. Using genome‐wide SNP data, we compared *Bd*‐naïve and *Bd*‐endemic populations and two 11‐year temporal resampling contrasts. Candidate SNPs were annotated to identify genomic context, nearby genes, putative functions and overlap with previously reported *Bd*‐associated transcriptomic responses. Using an empirical 99th‐percentile XtX threshold, scans identified high‐differentiation candidate SNPs in all contrasts. Most occurred in noncoding and putatively regulatory regions, with relatively few canonical immune genes. Candidate‐associated genes were more often linked to signalling, transcriptional regulation and cell‐cycle processes. A descriptive comparison with previous work in the species identified 64 candidate‐associated genes matching *Bd*‐associated transcriptomic annotations, supporting several immune‐adjacent, regulatory, cellular and reproduction candidates. Persistence under endemic *Bd* might reflect pathogen‐associated selection, compensatory recruitment and demographic change following recurrent adult mortality rather than uniform adaptive rescue through immune resistance.

## 1 Introduction

Understanding whether threatened wildlife populations can adapt to emerging stressors is a central question in biological conservation. Advances in population genomics now allow these signatures to be detected at fine scales, linking ecological pressures to molecular responses and providing a way to evaluate adaptive capacity in wild populations (Booker et al. 2017; Gautier 2015; Hoban et al. 2016; Mcmahon et al. 2014; Stapley et al. 2010). Studies across diverse taxa have identified loci under selection associated with a wide variety of pressures including environmental gradients (Carbeck et al. 2023; Jones et al. 2012; Tiffin and Ross‐Ibarra 2014), salinity tolerance in fishes (Velotta et al. 2022; Yang et al. 2025; Yu et al. 2022), high‐altitude adaptation in mammals (Friedrich and Wiener 2020; Lyu et al. 2022; Zhu et al. 2018), reproductive timing in birds (Bourret and Garant 2015; Laine et al. 2019; Verhagen et al. 2019) and migratory behaviour in salmonids (Tigano and Russello 2022). Collectively, these investigations show that adaptive responses are rarely restricted to a single trait or gene family but instead span multiple biological processes and may often reflect polygenic architectures distributed across many loci (Barghi et al. 2020).

Detecting genetic signatures of selection is not only important for understanding evolutionary history, but also for predicting the capacity of populations to persist under rapid environmental change (Green et al. 2022; Hoffmann et al. 2021). As persistent stressors accelerate the pace of change, studies that link genetic variation to ecological challenges provide a powerful framework for conservation. They reveal the traits and biological processes most likely to underpin adaptation and can inform management by identifying variation in adaptive potential among populations (Allendorf et al. 2010). Thus, investigating which genes or genomic regions are under selection in wild populations is critical for both evolutionary biology and applied conservation.

Among the strongest selective forces in nature are pathogens, which can leave strong and sometimes highly distributed signatures across host genomes (Barreiro and Quintana‐Murci 2010; Quintana‐Murci 2019). Infectious disease can impose severe and recurrent mortality, leading to rapid shifts in host allele frequencies at loci involved with pathogen recognition, immune activation or other traits that influence infection outcomes (Gaczorek et al. 2023; Savage and Zamudio 2011; Spurgin and Richardson 2010). However, persistence under endemic disease does not necessarily require increased resistance. Hosts can persist through tolerance, altered life history, demographic compensation, behavioural shifts or other host‐associated mechanisms that reduce infection costs or exposure (Grogan et al. 2023; Richards‐Zawacki 2010; Rowley and Alford 2013; Scheele et al. 2015). Pathogens might also influence reproduction in response to increased mortality due to disease (Brannelly et al. 2025; Katlav et al. 2022; Subramaniam et al. 2025), alter social or spatial behaviours that reduce pathogen exposure (Behringer et al. 2006; Stockmaier et al. 2021) and/or shape physiological traits such as fever responses in ectotherms that help eliminate infection (Haddad et al. 2023; Richards‐Zawacki 2010; Rowley and Alford 2013). Despite this potential, relatively few studies have directly demonstrated evolutionary change in wild populations following disease outbreaks, in part because sudden severe declines can make comparative persistence difficult to study.

Amphibian chytridiomycosis, caused by *Batrachochytrium dendrobatidis* (*Bd*), provides one of the clearest opportunities to examine these dynamics because it is among the most severe infectious diseases documented for vertebrate biodiversity (Scheele et al. 2019; Skerratt et al. 2007). *Bd* affects the host by disrupting skin function and ion balance, which in severe cases results in cardiac arrest and mortality (Berger et al. 2016; Campbell et al. 2012; Voyles et al. 2011). Since its emergence in the late 1970s (Berger et al. 1998, 2016), *Bd* has spread to more than 80 countries and infected over 700 amphibian species. It has caused many extinction events and been implicated as the main cause of decline in over 500 species (Castro Monzon et al. 2020; Olson et al. 2013; Scheele et al. 2019; Skerratt et al. 2007). Yet some affected populations persist or rebound in the presence of the pathogen (Knapp et al. 2016; Kupferberg et al. 2022; Scheele et al. 2017). How these populations continue to survive, despite persistent disease pressure, remains an important focus of amphibian research.

Most genomic work on amphibian responses to *Bd* has emphasized immune function, particularly variation at MHC loci, where certain alleles are associated with reduced infection loads or higher survival (Fu 2024; Kosch et al. 2016; Savage and Zamudio 2011; Spurgin and Richardson 2010; Trujillo et al. 2021). Direct genome‐scale tests in wild populations are still uncommon, and comparative transcriptomic studies indicate that responses to *Bd* vary among susceptible and resistant species. Resistant species can maintain skin integrity, whereas susceptible species may show weak or downregulated immune and skin‐associated responses or vigorous immune‐gene upregulation that nonetheless fails to prevent disease (Ellison et al. 2014; Eskew et al. 2018; Rosenblum et al. 2012). Adding to this, a study of resistant Japanese *Rana* frogs with a likely long evolutionary relationship with *Bd* found toll‐like receptor genes (TLR2 and TLR4) are mostly conserved, with just a few positions showing signs of adaptation and generally low expression, suggesting fine‐tuned and site‐specific change rather than broad shifts (Lau et al. 2018). Whole‐exome data from Panamanian harlequin frogs comparing pre‐ and post‐*Bd* samples reported no clear directional shifts at putative *Bd*‐resistance genes but did identify a shortlist of other candidate loci (Byrne et al. 2021). Most recently, a comparison of *Bd*‐naïve and recovering populations of *Rana sierrae* identified genomic variants associated with recovery, including candidates related to skin integrity, intracellular regulation and interferon‐associated processes, while also finding no evidence that recovery was driven by a single enriched functional pathway (Byrne et al. 2026). Beyond these few examples, relatively little work has explicitly tested whether endemic *Bd* leaves repeatable population‐genomic signatures across spatial and temporal contrasts in wild amphibians.

Building on this gap, the alpine tree frog (*Litoria verreauxii alpina*) offers a well‐studied case of persistence under *Bd* endemism (Brannelly, McCallum, et al. 2021). In this species, continued survival appears driven by reproduction and recruitment of scattered small populations at artificial permanent water bodies despite high disease‐related adult mortality (Brannelly et al. 2015; Scheele et al. 2015). A large proportion of juveniles survive to breeding age at approximately 2 years old, but individuals commonly become infected during the breeding season, after which most die. As a result, populations that historically included longer‐lived adults, with individuals living up to approximately 7 years, are now maintained largely through early‐life survival and recruitment into the breeding population (Scheele et al. 2015). Infection with *Bd* appears to elevate reproductive output, with both males and females increasing breeding effort when infected (Brannelly et al. 2016), and there is evidence that long‐term endemism has increased baseline sperm production in males (Brannelly, McCallum, et al. 2021; Brannelly, Webb, et al. 2021). Although immune activity rises with *Bd* infection, this heightened response does not seem protective (Grogan, Mulvenna, et al. 2018). Together, these findings suggest that persistence in this system might be shaped less by classic immune resistance and more by altered selective pressures on demography, recruitment, and reproduction under recurrent adult mortality. Fifteen years of work on the species' ecology, demography and disease dynamics provide a strong foundation and now pave the way for genome‐scale investigations in wild populations.

Genomic resources for *L. v. alpina*, including a reference genome and transcriptome (Wendt and Brannelly et al. 2025), now make it possible to test directly whether loci across the genome show signatures consistent with pathogen‐driven differentiation, while also building on the broader expansion of amphibian genomic resources in recent years (Kosch et al. 2024). Here, we use genome‐wide SNP data from both spatial exposure contrasts (*Bd*‐naïve versus endemic) and temporal resampling within populations to test whether persistent *Bd* exposure is associated with detectable and potentially repeatable signatures of genomic differentiation in wild *L. v. alpina* populations. Specifically, we ask whether candidate loci are concentrated in canonical immune genes commonly implicated in amphibian responses to *Bd*, including MHC loci, pattern‐recognition receptors such as Toll‐like receptors, cytokine‐ and interferon‐associated genes and other immune‐related genes. We contrast this expectation with the possibility that candidate loci are distributed across broader regulatory, cellular or reproduction related processes. We also examine whether similar signals recur across independent contrasts because repeatable genomic signatures would be expected if similar pathogen‐mediated selection acted across populations and time. Conversely, weak overlap among contrasts would indicate that persistence is associated with population‐specific responses, diffuse polygenic differentiation or genomic effects associated with demographic and life‐history shifts under endemic *Bd*. It is therefore possible that genomic differentiation reflects this *Bd*‐driven shift in life history, whereby high adult mortality truncates age structure and increases reliance on young breeding cohorts rather than, or in addition to, direct adaptation to the pathogen itself (Heard et al. 2024; Scheele et al. 2016). This study contributes to the still limited body of work examining pathogen‐associated genomic change in amphibians. In addition to clarifying how endemic disease may shape genomic variation in this species, the candidate loci identified here provide a foundation for future comparative and functional studies in amphibians, including tests of whether similar regulatory, immune or reproduction related processes are repeatedly implicated across *Bd*‐affected taxa.

## 2 Materials and Methods

### 2.1 Study System and Samples

Samples were collected from 9 total sites (2 sites were sampled twice, 11 years apart) from both New South Wales (NSW: sites A‐D and N1) and Victoria (VIC; Sites X‐Z and N2) populations (Figure 1). Two of the 9 sites were *Bd‐naïve* at the timing of sampling, while the other sites had a long exposure history with the disease, at least 20 years at the time of sampling and are referred to as disease endemic. Toe clips from individuals from naïve population N2 (2013) were acquired from the Museums Victoria tissue and DNA collection and kept at −80°C. All other samples (2013, 2018 and 2024) were collected as toe clips and stored at −80°C (Brannelly, Hunter, et al. 2016; Brannelly et al. 2025). *Bd*‐naïve status is supported by continued non‐detection in later surveys, with N1 remaining *Bd*‐negative in subsequent monitoring (Brannelly, Hunter et al. 2016) and N2 likewise remaining *Bd*‐negative in later surveys (Banks et al. 2020). A full list of sample populations as well as exposure status can be found in Table 1.

**FIGURE 1:**
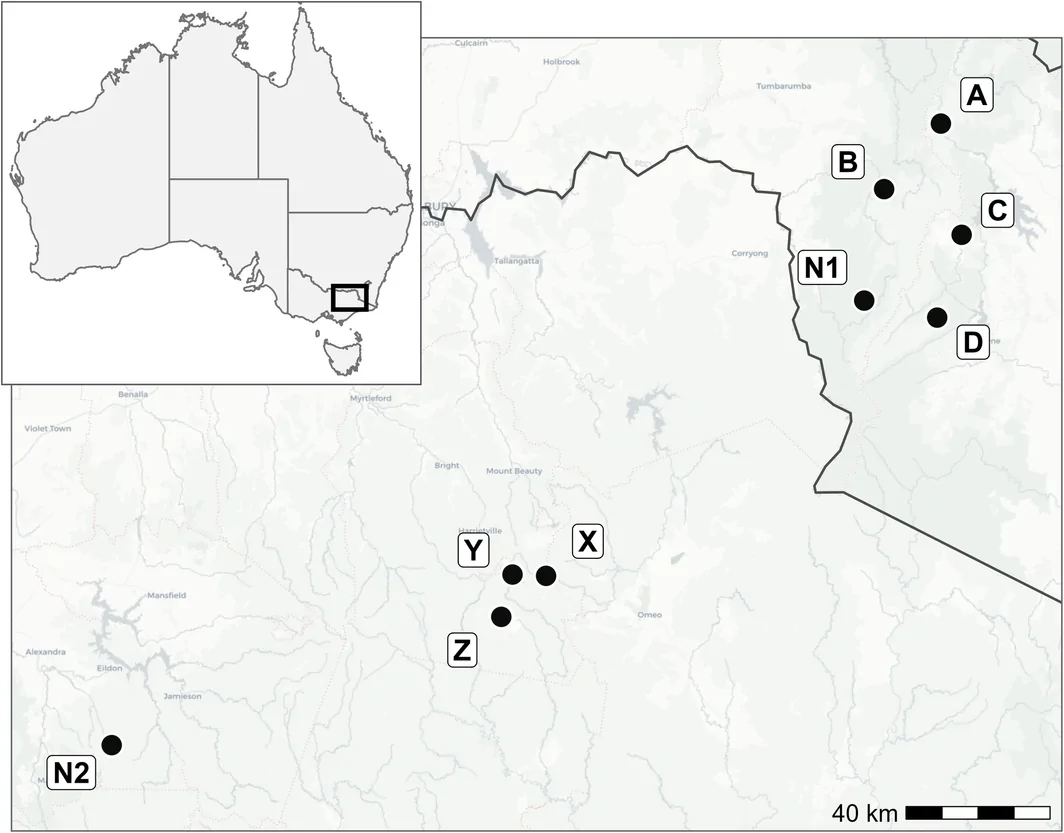
Map of sampled *Litoria verreauxii alpina* populations in southeastern Australia. Black circles indicate population localities, with labels corresponding to population codes used throughout the study.

**TABLE 1:** Summary of sampled populations, including collection year, state, *Batrachochytrium dendrobatidis* (*Bd*) exposure status and sample size (*N*).

Population	Year	State	*Bd* exposure status	*N*
N1	2013	NSW	Naïve	66
N2	2013	VIC	Naïve	10
X	2018	VIC	Endemic	54
Y	2018	VIC	Endemic	41
Z	2018	VIC	Endemic	197
A	2013	NSW	Endemic	43
A	2024	NSW	Endemic	23
B	2024	NSW	Endemic	23
C	2013	NSW	Endemic	43
C	2024	NSW	Endemic	24
D	2024	NSW	Endemic	24

*Note:* Populations are classified as either Naïve (not previously exposed to *Bd* at time of sampling) or Endemic (historically exposed to *Bd*). Samples were collected across New South Wales (NSW) and Victoria (VIC) from 2013 to 2024.

### 2.2 Sequencing and Variant Discovery

Tissue samples were sent to Diversity Arrays Technology (DArT; Canberra, Australia) for genotyping using the DArTseq platform. DArTseq is a reduced‐representation sequencing approach that combines restriction enzyme‐based genome complexity reduction with next‐generation sequencing to identify single nucleotide polymorphisms (SNPs) and presence/absence variants (PAVs) across the genome (Cruz et al. 2013; Jaccoud et al. 2001; Kilian et al. 2012; Sansaloni et al. 2011). This approach was chosen because amphibians often have large and repetitive genomes, making whole‐genome resequencing costly for population‐level sampling. DArTseq was also appropriate because this project was initiated before a species‐specific reference genome was available and reduced‐representation sequencing can generate genome‐wide SNP markers without requiring a reference genome for initial marker discovery. DArTseq provides a cost‐effective method for surveying thousands of genome‐wide loci across many individuals and is designed to enrich for reproducible, low‐copy markers while reducing representation of highly repetitive genomic regions. Genomic DNA was digested using the restriction enzymes Pstl and Sphl to target a consistent subset of low‐copy genomic regions, after which adapters were ligated to the resulting fragments and the libraries were PCR‐amplified. The amplified fragments were then sequenced on an Illumina HiSeq2500 platform. Sequence reads were processed through DArT's proprietary analytical pipeline to identify high‐confidence SNP and PAV markers. The DArT pipeline includes demultiplexing, read clustering, marker discovery, genotype calling and marker‐level quality filtering based on reproducibility, call rate and polymorphism information content. Specific internal thresholds for read depth, mapping quality and indel handling are proprietary to DArT and were not independently modified. Marker‐level read depth was summarized from DArT‐provided AvgCountRef and AvgCountSnp values as AvgCountRef + AvgCountSnp; these columns represent average tag read counts across samples with non‐zero tag counts for the reference and alternate allele rows, respectively. Per‐individual read‐depth values were not included in the standard DArT SNP output. SNP positions were assigned by aligning SNP‐containing sequence tags to the *Litoria verreauxii alpina* reference genome (Wendt and Brannelly et al. 2025). Subsequent filtering steps applied to each dataset and comparison are described below for relevant analyses.

### 2.3 Candidate SNPs Showing Elevated Differentiation

To identify single nucleotide polymorphisms (SNPs) showing elevated differentiation potentially consistent with pathogen‐associated selection related to *Bd* exposure or temporal changes in population genetic structure, we performed genome‐wide selection scans using the core model implemented in BayPass version 3.0 (Gautier 2015; Olazcuaga et al. 2020). BayPass is a Bayesian hierarchical model that estimates locus‐specific differentiation (XtX) while accounting for population covariance structure, thereby improving robustness to demographic history and population non‐independence. This approach is well‐suited to detecting loci with unusually high differentiation that may be under divergent or directional selection, particularly in datasets spanning both spatial and temporal contrasts. We used the BayPass core model because our analyses were designed as differentiation scans across predefined allele‐count contrasts.

We constructed two different contrasts for the selection scan: (1) a grouping based on population *Bd* exposure status at time of sampling, and (2) temporal comparisons within two long‐term populations. For the *Bd* exposure contrast, populations N1 and N2 were designated as *Bd*‐naive based on sampling dates preceding the earliest confirmed detection of *Bd* in these populations. All other populations in the dataset were classified as *Bd*‐endemic. For this exposure contrast, allele counts were pooled within the two exposure groups prior to BayPass analysis.

To ensure high‐quality data for the selection scans, we applied tailored filtering steps to each grouping using the *dartR* package (Gruber et al. 2018; Mijangos et al. 2022) in R (R Core Team 2025) within the RStudio interface (Posit Team 2025). For the comparison between *Bd*‐naïve and *Bd*‐endemic populations, we removed loci with call rates below 95% across individuals and excluded individuals with genotype call rates below 85%. A slightly less stringent filtering threshold was used for this contrast due to the greater evolutionary divergence and temporal spread between populations, which reduced the number of shared high‐quality loci available. Applying relaxed thresholds ensured sufficient SNP retention for downstream analyses. In contrast, for each of the two temporal comparisons (A_2013 vs. A_2024 and C_2013 vs. C_2024), we applied a locus‐level call rate threshold of 95% and filtered individuals with call rates below 90%. In all cases, loci with entirely missing genotypes across all individuals were removed. Filtering was performed separately for each grouping to maximize the retention of high‐confidence loci specific to each contrast.

For each SNP, we calculated the total number of reference and alternate alleles within each group by summing allele dosage values across individuals, effectively generating group‐level allele count matrices. Reference allele counts were computed as 2—dosage, while alternate allele counts were taken directly from the dosage matrix. The final BayPass input file was constructed by interleaving the reference and alternate allele counts for each group, yielding a two‐column‐per‐group format consistent with BayPass requirements.

BayPass was executed using the core model with two allele‐count groups specified. The model was run with the default Markov Chain Monte Carlo (MCMC) sampling scheme, which consisted of three phases: (i) 20 pilot runs of 500 iterations each to adjust the proposal distributions, (ii) a burn‐in period of 5000 iterations and (iii) final sampling of 1000 parameter values drawn every 20 iterations (totalling 20,000 iterations). This configuration balances convergence accuracy with computational efficiency for moderate‐size datasets. The primary output of interest was the XtX statistic, which quantifies locus‐specific differentiation while incorporating the estimated Ω (omega) matrix, a group covariance matrix inferred from genome‐wide allele frequency covariation. SNPs with high XtX values relative to the empirical distribution were interpreted as candidate loci potentially associated with divergent selection. In addition to the *Bd* comparison, we performed temporal selection scans within two populations that were sampled at two different time points. For site A (A_2013 vs. A_2024) and site C (C_2013 vs. C_2024), we constructed separate two‐group contrasts using the same allele counting and formatting approach as described above. This allowed detection of SNPs showing allele frequency shifts over time, which may reflect evolutionary responses to *Bd* or other environmental changes. Each temporal dataset was processed and analyzed independently using the BayPass core model with two allele‐count groups. For all three contrasts, candidate loci were defined as SNPs within the upper 1% of the empirical XtX distribution within that contrast. This threshold was selected to identify a focused set of the most strongly differentiated loci within each analysis for downstream annotation. Candidate loci were interpreted as putative genomic regions of interest consistent with elevated differentiation. We then filtered out all SNPs with unknown scaffold positions before proceeding to the next step.

### 2.4 Predicting SNP Effects

We annotated the functional effects of each SNP using SnpEff v5.2 (Cingolani et al. 2012), executed through the Galaxy Australia platform (https://usegalaxy.org.au). We supplied SnpEff with the custom reference genome and corresponding GFF3 annotation file for *Litoria verreauxii alpina*, which included curated transcript models (Wendt and Brannelly et al. 2025). SnpEff classified each SNP based on its position relative to annotated genomic features. Variants were categorized as falling within coding sequence, introns or in flanking regions located within 1000 base pairs upstream or downstream of annotated genes. Intergenic SNPs were annotated with the nearest upstream and downstream genes, where possible. Gene identifiers and genomic coordinates provided by SnpEff were used to link each SNP to its associated protein‐coding region or nearest gene for downstream functional analysis.

SnpEff outputs were parsed in R to extract gene identifiers and the positional classification of each SNP (e.g., intergenic, intronic, coding and upstream/downstream). Using these gene identifiers, we matched each SNP to its corresponding protein sequence from the *Litoria verreauxii alpina* transcriptome. Protein sequences were retrieved from a FASTA file and linked to their respective gene IDs, allowing us to append amino acid sequences to the SNP annotation table.

We then generated a FASTA file containing all unique protein sequences for functional analysis. Protein homology searches were performed using BLASTp against the clustered non‐redundant (clusteredNR) protein database via the NCBI web server (Camacho et al. 2009). The top match for each gene was extracted and parsed in R to obtain relevant information, including cluster ancestor, percent identity, query coverage, alignment length, e‐value and representative sequence. These results were merged with the annotated SNP dataset to produce a comprehensive table linking genomic variation to predicted function. Candidate‐associated genes were assigned to broad functional categories based on SnpEff genomic context, BLASTp protein descriptions, RefSeq or NCBI functional annotations, and manual review of known gene functions. To compare candidate‐associated genes with a previous experiment examining *Bd*‐associated transcriptomic responses involving *L. v. alpina* collected from four populations in New South Wales, we compared candidate annotations with liver, skin and spleen transcriptome annotations reported by Grogan, Mulvenna, et al. (2018). Candidate‐associated genes were matched to Grogan et al. annotations using exact accession identifiers where available and standardized protein descriptions. Matches were summarized descriptively because the datasets differed in sequencing approach, tissue type and analytical design. A searchable spreadsheet containing annotated candidate loci is provided as Data S1.

## 3 Results

### 3.1 SNP Quality, Filtering, and Genomic Distribution

The DArT‐provided SNP dataset, following DArT's proprietary marker discovery and initial quality filtering, contained 72,060 SNP loci before downstream analysis‐specific filtering in this study. Marker‐level read depth, estimated from DArT‐provided AvgCountRef and AvgCountSnp values as AvgCountRef + AvgCountSnp, averaged 13.88 reads per SNP locus (median = 12.37; interquartile range = 9.42–16.66; range = 5.01–68.90). These values represent marker‐level read‐count summaries rather than per‐individual sequencing depth, which was not included in the standard DArT SNP output. Mean marker reproducibility in the full imported dataset was high (mean RepAvg = 0.995). As each selection scan involved a distinct subset of sampling groups, SNP filtering was conducted separately for each grouping to retain high‐confidence loci within each contrast. Individual‐level filtering removed one individual from the *Bd*‐naïve versus endemic exposure contrast and one individual from the site C temporal comparison; no individuals were removed from the site A temporal comparison. Consequently, the number of SNPs analysed varied by dataset, with 5440 SNPs retained for the *Bd* naïve versus endemic exposure contrast, 4775 for the site A temporal comparison (2013 vs. 2024) and 5103 for the site C temporal comparison (2013 vs. 2024). Because each contrast retained a different SNP set, XtX values and candidate loci were interpreted within each analysis rather than compared directly across contrasts.

To evaluate genome‐wide marker distribution, retained SNP counts and SNP density per Mb were summarized by scaffold for the DArT‐provided SNP dataset and for each contrast (Table 2). In the DArT‐provided dataset, 52,050 SNPs were positioned on scaffolds 1–12, with scaffold‐level densities ranging from 18.38 to 22.33 SNPs/Mb. In the analysis‐specific datasets, positioned SNPs were distributed across all 12 scaffolds, with scaffold‐level densities ranging from 1.18 to 2.14 SNPs/Mb in the *Bd*‐naïve versus endemic exposure contrast, 0.96 to 1.74 SNPs/Mb in the site A temporal comparison, and 1.04 to 1.84 SNPs/Mb in the site C temporal comparison. Although absolute SNP counts were higher on larger scaffolds, scaffold‐standardized densities indicated broad genome‐wide marker distribution rather than concentration on a single scaffold.

**TABLE 2:** Genome‐wide distribution of retained SNPs by scaffold for the DArT‐provided SNP dataset and each BayPass contrast.

Dataset/contrast	Total SNPs	Positioned SNPs (scaffolds 1–12)	Positioned SNPs (%)	Scaffold	Retained SNP count on scaffold	SNPs per Mb
DArT‐provided SNP dataset	72,060	52,050	72.2314738	1	10,769	20.609
				2	8527	21.124
				3	6000	21.540
				4	5572	20.862
				5	4886	22.325
				6	3944	19.524
				7	3446	18.376
				8	3344	20.327
				9	1834	20.063
				10	1531	19.379
				11	1414	19.023
				12	783	21.427
Bd‐naive vs. endemic exposure contrast	5440	4722	86.8014706	1	976	1.868
				2	754	1.868
				3	597	2.143
				4	416	1.558
				5	420	1.919
				6	390	1.931
				7	359	1.914
				8	342	2.079
				9	162	1.772
				10	115	1.456
				11	148	1.991
				12	43	1.177
Site A temporal comparison	4775	3907	81.8219895	1	796	1.523
				2	600	1.486
				3	484	1.738
				4	380	1.423
				5	353	1.613
				6	333	1.648
				7	287	1.530
				8	283	1.720
				9	141	1.542
				10	103	1.304
				11	112	1.507
				12	35	0.958
Site C temporal comparison	5103	4016	78.6988046	1	782	1.497
				2	651	1.613
				3	513	1.842
				4	362	1.355
				5	375	1.713
				6	330	1.634
				7	305	1.626
				8	293	1.781
				9	141	1.542
				10	108	1.367
				11	118	1.587
				12	38	1.040

*Note:* The DArT‐provided SNP dataset represents SNPs retained after DArT's proprietary marker discovery and initial quality filtering, before downstream analysis‐specific filtering in this study. Positioned SNPs are those assigned to scaffolds 1–12 of the *Litoria verreauxii alpina* reference genome. For each dataset or contrast, retained SNP counts are shown by scaffold, and SNP density was calculated as retained SNP count per Mb using reference scaffold lengths.

### 3.2 Candidate SNPs Showing Elevated Differentiation

Genome‐wide selection scans using the BayPass core model identified SNPs with elevated XtX values, indicative of potential divergent or directional selection. Candidate loci were defined as those exceeding the 99th percentile of the empirical XtX distribution within each analysis. Using this threshold, the *Bd* naïve versus endemic exposure contrast yielded 50 high‐XtX SNPs, suggesting loci potentially associated with disease history or pathogen‐mediated selection. The site A temporal comparison identified 41 candidate SNPs showing allele frequency shifts across time, while the site C temporal comparison revealed 40 candidate SNPs with temporal allele frequency shifts. These candidate loci represent the upper tail of the XtX distribution relative to the full set of analysed SNPs in each comparison and should therefore be interpreted as putative outliers rather than formally significant selected loci. For each comparison, genome‐wide XtX values were plotted using Manhattan plots, where all retained SNPs located between chromosomes 1–12 were displayed, with the empirical 99th percentile XtX threshold overlaid to identify candidate outlier loci (Figure 2).

**FIGURE 2:**
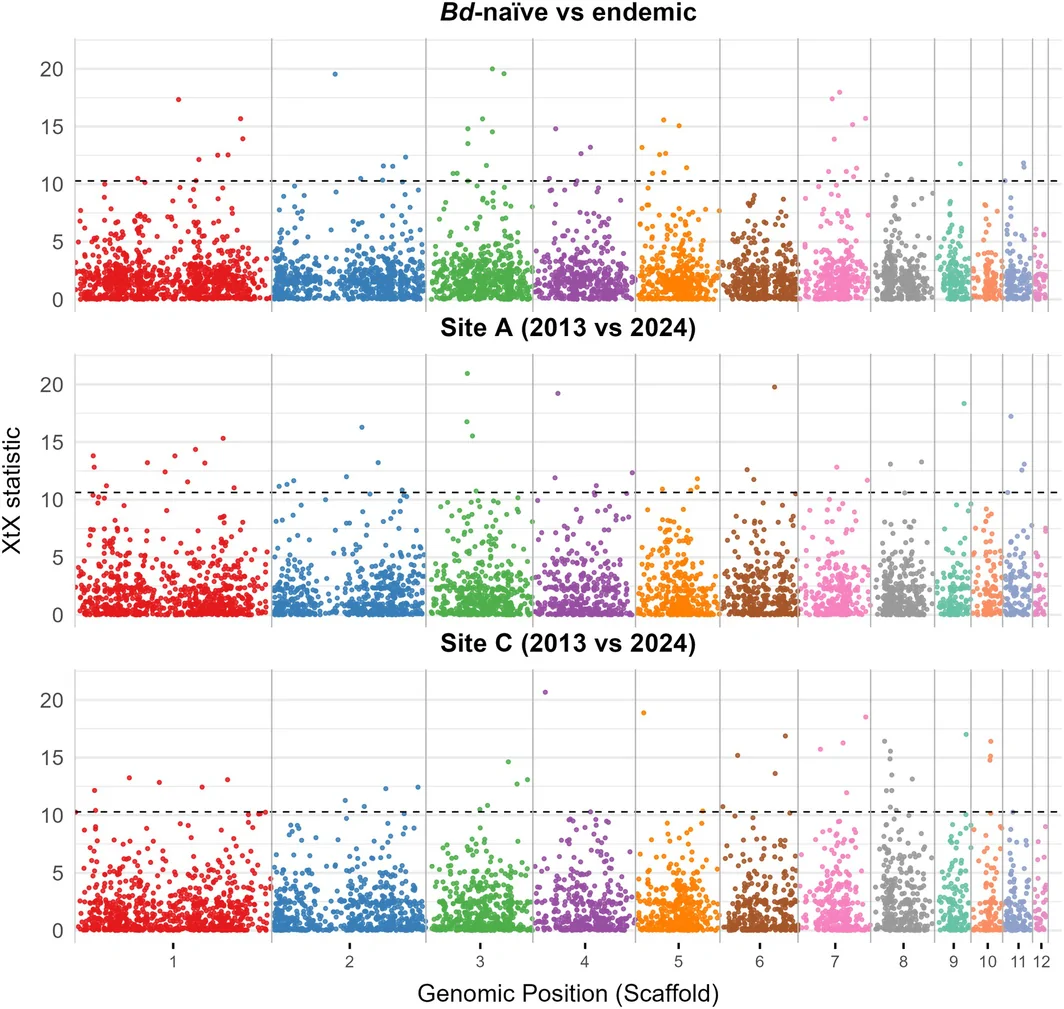
Genome‐wide distribution of XtX statistics across 12 major scaffolds in *Litoria verreauxii alpina*, identifying SNPs under potential selection. From top to bottom: Comparison between *Batrachochytrium dendrobatidis* (*Bd*) naïve and *Bd* endemic populations; Temporal comparison within site A (2013 vs. 2024); Temporal comparison within site C (2013 vs. 2024). Each point represents a single SNP, plotted according to its genomic position (x‐axis) and XtX statistic (y‐axis), a measure of population differentiation estimated using BayPass. Colours indicate separate scaffolds with scaffold widths representing relative size. The dashed horizontal line indicates the empirical 99th percentile XtX threshold used to define candidate outlier loci.

### 3.3 Functional Annotation and Overlap Among Contrasts

Functional annotation of candidate loci was performed using SnpEff v5.2. Initial parsed annotation labels identified 13 SNPs as occurring within annotated gene spans (‘Gene’) and 19 SNPs as intronic. However, coordinate‐based validation against the reference GFF3 showed that all 13 SNPs labelled as ‘Gene’ also fell within intronic regions of annotated gene spans, while 18 of the 19 SNPs labelled as Intron were confirmed as intronic and one overlapped a coding sequence (CDS). A total of 3 variants were located within 1 kb of gene boundaries and were classified as upstream or downstream of known genes. The majority of SNPs (74%) were located in intergenic regions and were annotated with the nearest flanking genes (Table 3). These intergenic variants may still be of functional relevance if they influence gene regulation or expression.

**TABLE 3:** Annotation of candidate genes associated with outlier SNPs identified across the *Bd*‐endemic, A temporal and C temporal comparisons.

Scaffold	Gene	SNP location	Population	Classification	Cluster ancestor	Representative sequence	Query length	Query cover	Evalue	% Identity
1	799	49206631	A	Intergenic	*Bufotes viridis*	CAK8599427.1 Ras GTPase‐activating‐like protein IQGAP1 [ *Bufotes viridis* ]	617	94	0	93.24
1	800	49206631	A	Intergenic	Anura	XP_069818872.1 synaptic vesicle glycoprotein 2C [ *Dendropsophus ebraccatus* ]	326	100	0	89.66
1	822	51811604	A	Intergenic	*Hyla sarda*	XP_056395945.1 beta‐hexosaminidase subunit beta isoform X1 [ *Hyla sarda* ]	464	98	0	85.24
1	823	51811604	A	Intergenic	*Hemicordylus capensis*	XP_053151124.1 ectoderm‐neural cortex protein 1 isoform X1 [ *Hemicordylus capensis* ]	589	100	0	93.21
1	832	52826644	C	Intergenic	NA	NA	NA	NA	NA	NA
1	833	52826644	C	Intergenic	Cellular organisms	NP_000973.2 large ribosomal subunit protein eL21 [ *Homo sapiens* ]	159	100	1.00E‐98	86.25
1	867	55463684	C	Intergenic	Hyloidea	XP_044131961.1 dihydrofolate reductase [ *Bufo gargarizans* ]	119	60	5.00E‐34	85.92
1	868	55463684	C	Intergenic	*Eleutherodactylus coqui*	XP_066459044.1 DNA mismatch repair protein Msh3 [ *Eleutherodactylus coqui* ]	321	77	4.00E‐123	75.59
1	1246	84335740	A	Intergenic	*Eleutherodactylus coqui*	XP_066459356.1 GPN‐loop GTPase 3 [ *Eleutherodactylus coqui* ]	284	100	0	96.48
1	1247	84335740	A	Intergenic	Tetrapoda	NP_001080054.1 Actin‐related protein 2/3 complex subunit 3‐B [ *Xenopus laevis* ]	178	100	2.00E‐124	93.82
1	1943	145029745	C	Intergenic	*Bufotes viridis*	CAK8601969.1 Ig mu chain C region secreted form [ *Bufotes viridis* ]	434	99	0	65.66
1	1944	145029745	C	Intergenic	*Bufo bufo*	XP_040272778.1 uncharacterized protein LOC120989007 isoform X1 [ *Bufo bufo* ]	1373	97	0	56.85
1	2196	167616714	*Bd*	Intergenic	Bufonidae	XP_040273015.1 uncharacterized protein C9orf85 homologue [ *Bufo bufo* ]	164	99	2.00E‐82	75.9
1	2197	167616714	*Bd*	Intergenic	NA	NA	NA	NA	NA	NA
1	2496	192880379	A	Intergenic	Hyloidea	XP_069819593.1 protein AF‐9 [ *Dendropsophus ebraccatus* ]	143	83	9.00E‐72	95.76
1	2497	192880379	A	Intergenic	Hyloidea	XP_069819593.1 protein AF‐9 [ *Dendropsophus ebraccatus* ]	226	89	2.00E‐112	95.52
1	2867	224541033	C	Intergenic	*Xenopus tropicalis*	CAL49416.1 PC4 and SFRS1 interacting protein 1 partial [ *Xenopus tropicalis* ]	102	94	4.00E‐63	95.83
1	2868	224541033	C	Intergenic	NA	NA	NA	NA	NA	NA
1	3056	239757087	A	Intergenic	*Biomphalaria glabrata*	KAI8793994.1 hypothetical protein BgiBS90_004370 [ *Biomphalaria glabrata* ]	831	99	1.00E‐60	38.1
1	3057	239757087	A	Intergenic	*Heterodontus francisci*	XP_067896123.1 nascent polypeptide‐associated complex subunit alpha muscle‐specific form‐like [ *Heterodontus francisci* ]	1902	98	7.00E‐86	24.87
1	3370	265813852	A	Intergenic	*Camelus dromedarius*	KAB1266904.1 Neurexin‐1 [ *Camelus dromedarius* ]	179	88	6.00E‐110	98.1
1	3371	265813852	A	Intergenic	Neobatrachia	XP_073528215.1 neurexin‐1 isoform X10 [ *Phyllobates terribilis* ]	372	95	0	96.07
1	3480	275534375	*Bd*	Intergenic	*Engystomops pustulosus*	KAG8584286.1 hypothetical protein GDO81_008764 [ *Engystomops pustulosus* ]	200	100	4.00E‐121	82.61
1	3481	275534375	*Bd*	Intergenic	*Bufotes viridis*	CAK8635511.1 Transcriptional‐regulating factor 1 [ *Bufotes viridis* ]	393	70	4.00E‐165	88.36
1	3871	299464343	A	Intron	*Ranitomeya imitator*	CAJ0939192.1 unnamed protein product [ *Ranitomeya imitator* ]	168	96	3.00E‐107	93.83
1	4298	320343605	A	Intergenic	*Phyllobates terribilis*	XP_073511428.1 formyl peptide receptor 2‐like [ *Phyllobates terribilis* ]	122	98	4.00E‐18	39.5
1	4299	320343605	A	Intergenic	*Dendropsophus ebraccatus*	XP_069815763.1 uncharacterized protein [ *Dendropsophus ebraccatus* ]	295	52	1.00E‐32	45.51
1	4317	321682257	*Bd*	Intergenic	*Xenopus laevis*	OCT55537.1 hypothetical protein XELAEV_18001264mg [ *Xenopus laevis* ]	249	39	4.00E‐48	79.59
1	4318	321682257	*Bd*	Intergenic	NA	NA	NA	NA	NA	NA
1	4384	329347816	*Bd*	Intergenic	*Bufotes viridis*	CAK8654144.1 LINE‐1 retrotransposable element ORF1 protein [ *Bufotes viridis* ]	374	76	5.00E‐63	42.81
1	4385	329347816	*Bd*	Intergenic	NA	NA	NA	NA	NA	NA
1	4467	337896104	C	Intron	*Bufo gargarizans*	XP_044147357.1 uncharacterized protein LOC122935632 [ *Bufo gargarizans* ]	500	79	5.00E‐74	39.62
1	4546	345125785	A	Intron	Dendrobatinae	XP_069582033.1 beta‐taxilin [ *Ranitomeya imitator* ]	150	100	1.00E‐66	81.17
1	5011	379350360	*Bd*	Downstream	*Pseudophryne corroboree*	XP_063770585.1 gypsy retrotransposon integrase‐like protein 1 [ *Pseudophryne corroboree* ]	215	61	3.00E‐66	78.36
1	5227	393647967	A	Intergenic	*Hyperolius riggenbachi*	XP_068137431.1 testis development‐related protein [ *Hyperolius riggenbachi* ]	81	100	9.00E‐43	91.36
1	5228	393647967	A	Intergenic	Hyloidea	XP_040284464.1 testis development‐related protein isoform X1 [ *Bufo bufo* ]	119	97	1.00E‐73	96.52
1	5453	405421631	C	Intron	Neobatrachia	XP_066457701.1 replication factor C subunit 4 [ *Eleutherodactylus coqui* ]	363	99	0	92.52
1	5467	406702030	*Bd*	Intron	*Coturnix japonica*	XP_015727290.1 transcription factor SOX‐2 [ *Coturnix japonica* ]	314	100	0	93.97
1	5675	422681628	A	Downstream	*Engystomops pustulosus*	KAG8580962.1 hypothetical protein GDO81_007494 [ *Engystomops pustulosus* ]	361	98	0	90.91
1	5869	439907595	*Bd*	Intergenic	*Eleutherodactylus coqui*	XP_066445582.1 autoimmune regulator‐like [ *Eleutherodactylus coqui* ]	547	98	0	77.47
1	5870	439907595	*Bd*	Intergenic	*Dendropsophus ebraccatus*	XP_069831604.1 EF‐hand domain‐containing protein D1 [ *Dendropsophus ebraccatus* ]	96	100	1.00E‐45	85.57
1	5916	445849186	*Bd*	Intergenic	NA	NA	NA	NA	NA	NA
1	5917	445849186	*Bd*	Intergenic	Caprinae	XDA73022.1 hypothetical protein R6Z07_003346 [ *Ovis aries* ]	341	100	0	98.83
2	201	14569275	A	Intergenic	*Hyla sarda*	XP_056407734.1 uncharacterized protein LOC130303239 [ *Hyla sarda* ]	106	57	6.00E‐04	39.68
2	202	14569275	A	Intergenic	*Trinorchestia longiramus*	KAF2352654.1 Reverse transcriptase RNA‐dependent DNA polymerase [Trinorchestia longiramus]	578	97	1.00E‐129	39.06
2	2246	163046339	*Bd*	Intergenic	*Ranitomeya imitator*	CAJ0959241.1 unnamed protein product partial [ *Ranitomeya imitator* ]	419	75	5.00E‐158	73.9
2	2247	163046339	*Bd*	Intergenic	Tetrapoda	AAH33404.1 C11orf30 protein [ *Homo sapiens* ]	82	100	7.00E‐50	97.56
2	2725	189808288	C	Intergenic	*Ranitomeya imitator*	CAJ0940184.1 unnamed protein product [ *Ranitomeya imitator* ]	163	77	2.00E‐12	30.08
2	2726	189808288	C	Intergenic	*Ranitomeya imitator*	CAJ0940184.1 unnamed protein product [ *Ranitomeya imitator* ]	163	77	5.00E‐13	30.83
2	2798	193078352	A	Intron	*Hyla sarda*	XP_056417743.1 adiponectin‐like [ *Hyla sarda* ]	807	30	6.00E‐112	77.08
2	3306	230791113	*Bd*	Intron	*Hyla sarda*	XP_056409648.1 seven in absentia homologue 3‐like [ *Hyla sarda* ]	122	100	1.00E‐83	98.36
2	3370	234053799	A	Intergenic	Hyloidea	CAK8629099.1 Pcdh8 [ *Bufotes viridis* ]	1013	100	0	94.36
2	3371	234053799	A	Intergenic	*Chaetura pelagica*	KFU90818.1 hypothetical protein M959_14824 partial [ *Chaetura pelagica* ]	142	54	0.011	66.67
2	3466	240295176	C	Intron	NA	NA	NA	NA	NA	NA
2	3816	277098168	A	Intergenic	Bufonidae	CAK8629861.1 Toll‐like receptor 8 [ *Bufotes viridis* ]	1048	98	0	84.55
2	3817	277098168	A	Intergenic	*Rhinatrema bivittatum*	XP_029460201.1 ribose‐phosphate pyrophosphokinase 2 isoform X2 [ *Rhinatrema bivittatum* ]	114	100	9.00E‐77	100
2	3976	288959801	*Bd*	Intron	Hyloidea	KAG9492589.1 hypothetical protein GDO78_000864 partial [ *Eleutherodactylus coqui* ]	846	99	0	86.68
2	3982	290857950	*Bd*	Intergenic	Laurasiatheria	EFB13527.1 hypothetical protein PANDA_019488 partial [ *Ailuropoda melanoleuca* ]	87	54	4.00E‐22	93.62
2	3983	290857950	*Bd*	Intergenic	NA	NA	NA	NA	NA	NA
2	4069	297377988	C	Intergenic	Neobatrachia	XP_066431466.1 ras GTPase‐activating protein 4‐like isoform X1 [ *Eleutherodactylus coqui* ]	1021	55	0	81.16
2	4070	297377988	C	Intergenic	*Engystomops pustulosus*	KAG8587705.1 hypothetical protein GDO81_005758 [ *Engystomops pustulosus* ]	86	90	1.00E‐45	94.81
2	4288	315711286	*Bd*	Intergenic	*Antechinus flavipes*	XP_051833771.1 zinc finger and BTB domain‐containing protein 25 isoform X1 [ *Antechinus flavipes* ]	432	100	0	90.28
2	4289	315711286	*Bd*	Intergenic	NA	NA	NA	NA	NA	NA
2	4639	340688881	A	Intergenic	*Xenopus tropicalis*	KAE8580458.1 hypothetical protein XENTR_v10024436 [ *Xenopus tropicalis* ]	242	100	2.00E‐172	99.17
2	4640	340688881	A	Intergenic	*Engystomops pustulosus*	XP_071977364.1 voltage‐gated delayed rectifier potassium channel KCNH8‐like isoform X3 [ *Engystomops pustulosus* ]	1077	97	0	89.94
2	4777	349845723	*Bd*	Intergenic	Hyloidea	XP_069841294.1 lanosterol synthase [ *Dendropsophus ebraccatus* ]	222	100	9.00E‐142	77.49
2	4778	349845723	*Bd*	Intergenic	NA	NA	NA	NA	NA	NA
2	5206	382708752	C	Intergenic	*Hyla sarda*	XP_056391876.1 uncharacterized protein LOC130284931 isoform X3 [ *Hyla sarda* ]	219	100	1.00E‐127	77.63
2	5207	382708752	C	Intergenic	*Hyla sarda*	XP_056391876.1 uncharacterized protein LOC130284931 isoform X3 [ *Hyla sarda* ]	344	100	0	85.76
2	6108	35413550	A	Intergenic	NA	NA	NA	NA	NA	NA
2	6109	35413550	A	Intergenic	NA	NA	NA	NA	NA	NA
2	6422	53304705	A	Intergenic	NA	NA	NA	NA	NA	NA
2	6423	53304705	A	Intergenic	NA	NA	NA	NA	NA	NA
3	921	67041973	*Bd*	Intergenic	Bufonidae	CAK8618527.1 Fumarylacetoacetate hydrolase domain‐containing protein 2 [ *Bufotes viridis* ]	319	100	0	89.34
3	922	67041973	*Bd*	Intergenic	NA	NA	NA	NA	NA	NA
3	1069	77847009	*Bd*	Intergenic	Neobatrachia	XP_063781593.1 D‐glucuronyl C5‐epimerase isoform X1 [ *Pseudophryne corroboree* ]	961	61	0	90.66
3	1070	77847009	*Bd*	Intergenic	NA	NA	NA	NA	NA	NA
3	1393	103743877	A	Intergenic	*Hyla sarda*	XP_056428190.1 probable phospholipid‐transporting ATPase IM isoform X2 [ *Hyla sarda* ]	412	100	0	98.79
3	1394	103743877	A	Intergenic	*Eleutherodactylus coqui*	XP_066448726.1 tRNA‐uridine aminocarboxypropyltransferase 1 isoform X2 [ *Eleutherodactylus coqui* ]	270	74	1.00E‐115	83
3	1403	104830064	A	Intergenic	*Dendropsophus ebraccatus*	XP_069812705.1 SHC‐transforming protein 4 isoform X2 [ *Dendropsophus ebraccatus* ]	310	99	0	87.42
3	1404	104830064	A	Intergenic	*Dendropsophus ebraccatus*	XP_069839258.1 centrosomal protein of 152 kDa isoform X2 [ *Dendropsophus ebraccatus* ]	1758	99	0	78.24
3	1424	106306528	*Bd*	Intergenic	NA	NA	NA	NA	NA	NA
3	1424	106276673	*Bd*	Intron	NA	NA	NA	NA	NA	NA
3	1425	106306528	*Bd*	Intergenic	*Engystomops pustulosus*	XP_071995394.1 uncharacterized protein [ *Engystomops pustulosus* ]	287	93	3.00E‐46	37.28
3	1578	118403387	A	Intron	Anura	XP_056428543.1 neuronal acetylcholine receptor subunit alpha‐5‐like [ *Hyla sarda* ]	380	100	0	93.95
3	1680	128117341	A	Intergenic	Hyloidea	XP_056428740.1 CD276 antigen [ *Hyla sarda* ]	283	100	2.00E‐162	83.28
3	1681	128117341	A	Intergenic	*Bufotes viridis*	CAK8644258.1 LINE‐1 type transposase domain‐containing protein 1 [ *Bufotes viridis* ]	198	71	3.00E‐39	46.1
3	1786	138035333	C	Intron	Hyloidea	XP_072003716.1 rab‐like protein 2B isoform X1 [ *Engystomops pustulosus* ]	213	100	4.00E‐144	88.11
3	1909	145216568	*Bd*	Intergenic	Anura	NP_001005676.1 rab GDP dissociation inhibitor beta [ *Xenopus tropicalis* ]	446	100	0	92.38
3	1910	145216568	*Bd*	Intergenic	Bufonidae	CAK8620216.1 Ankyrin repeat domain‐containing protein 16 [ *Bufotes viridis* ]	296	98	0	86.94
3	2018	155386898	*Bd*	Intergenic	*Engystomops pustulosus*	XP_072003334.1 fibroleukin [ *Engystomops pustulosus* ]	422	100	0	88.6
3	2019	155386898	*Bd*	Intergenic	NA	NA	NA	NA	NA	NA
3	2050	158526605	C	Intergenic	*Engystomops pustulosus*	XP_072003273.1 coiled‐coil domain‐containing protein 71 L [ *Engystomops pustulosus* ]	210	100	2.00E‐119	86.92
3	2051	158526605	C	Intergenic	Anura	XP_063783122.1 phosphatidylinositol 45‐bisphosphate 3‐kinase catalytic subunit gamma isoform isoform X1 [ *Pseudophryne corroboree* ]	1107	100	0	93.13
3	2267	171205604	*Bd*	Intergenic	*Xenopus laevis*	OCT55537.1 hypothetical protein XELAEV_18001264mg [ *Xenopus laevis* ]	286	40	8.00E‐62	80.53
3	2267	171205604	*Bd*	Intergenic	*Xenopus laevis*	OCT55537.1 hypothetical protein XELAEV_18001264mg [ *Xenopus laevis* ]	286	40	8.00E‐62	80.53
3	2268	171205604	*Bd*	Intergenic	*Dendropsophus ebraccatus*	XP_069835539.1 protein kinase C delta type‐like [ *Dendropsophus ebraccatus* ]	227	43	1.00E‐21	57.58
3	2268	171205604	*Bd*	Intergenic	*Dendropsophus ebraccatus*	XP_069835539.1 protein kinase C delta type‐like [ *Dendropsophus ebraccatus* ]	227	43	1.00E‐21	57.58
3	2716	202010940	*Bd*	Intergenic	*Hyperolius riggenbachi*	XP_068128960.1 uncharacterized protein [ *Hyperolius riggenbachi* ]	295	100	2.00E‐110	62.62
3	2717	202010940	*Bd*	Intergenic	*Hyperolius riggenbachi*	XP_068128962.1 uncharacterized protein [ *Hyperolius riggenbachi* ]	134	92	3.00E‐61	74.64
3	2821	213662129	C	Intron	*Hyla sarda*	XP_056376039.1 uncharacterized protein LOC130273369 isoform X2 [ *Hyla sarda* ]	515	29	4.00E‐27	41.22
3	3188	236619427	C	Intergenic	*Prunus dulcis*	BBH02259.1 transposable element gene [ *Prunus dulcis* ]	354	100	4.00E‐103	44.09
3	3189	236619427	C	Intergenic	Bufonidae	CAK8623102.1 Solute carrier organic anion transporter family member 1A4 [ *Bufotes viridis* ]	415	79	0	90.18
3	3784	264307759	C	Intron	*Dendropsophus ebraccatus*	XP_069807883.1 poly(3‐hydroxybutyrate) depolymerase‐like isoform X2 [ *Dendropsophus ebraccatus* ]	298	100	0	86.87
4	386	27999205	C	Intron	*Pelobates cultripes*	CAH2282315.1 voltage‐dependent L‐type calcium channel subunit beta‐2 isoform X9 [ *Pelobates cultripes* ]	82	71	3.00E‐23	79.31
4	521	38733581	*Bd*	Intron	NA	NA	NA	NA	NA	NA
4	709	53762837	A	Intergenic	Bufo	XP_044150100.1 nucleoporin NUP42 [ *Bufo gargarizans* ]	448	100	0	71.61
4	710	53762837	A	Intergenic	*Engystomops pustulosus*	KAG8573161.1 hypothetical protein GDO81_012314 [ *Engystomops pustulosus* ]	597	100	0	78.44
4	723	55482534	*Bd*	Intergenic	*Bufo bufo*	XP_040289937.1 serine/threonine‐protein kinase 31 [ *Bufo bufo* ]	1083	82	0	68.93
4	724	55482534	*Bd*	Intergenic	*Ranitomeya imitator*	CAJ0958489.1 unnamed protein product [ *Ranitomeya imitator* ]	232	78	2.00E‐106	83.78
4	801	61377827	A	Intergenic	*Pristimantis euphronides*	CAN2389690.1 Transmembrane protein 108 [ *Pristimantis euphronides* ]	469	91	0	86.81
4	802	61377827	A	Intergenic	Anura	CAI9585138.1 unnamed protein product [ *Staurois parvus* ]	212	72	5.00E‐96	93.46
4	1333	111839784	*Bd*	Intergenic	*Bufo gargarizans*	XP_044134789.1 zinc finger MYM‐type protein 1‐like [ *Bufo gargarizans* ]	386	68	5.00E‐128	69.96
4	1334	111839784	*Bd*	Intergenic	NA	NA	NA	NA	NA	NA
4	1466	122613409	*Bd*	Intergenic	*Xenopus tropicalis*	AAI18825.1 gabbr2 protein partial [ *Xenopus tropicalis* ]	324	40	4.00E‐44	78.03
4	1467	122613409	*Bd*	Intergenic	NA	NA	NA	NA	NA	NA
4	1666	147561041	*Bd*	Intergenic	Anura	KAG9479283.1 hypothetical protein GDO78_012775 [ *Eleutherodactylus coqui* ]	227	100	1.00E‐163	98.68
4	1667	147561041	*Bd*	Intergenic	*Hyla sarda*	XP_056377055.1 ribonuclease P protein subunit p40 isoform X3 [ *Hyla sarda* ]	359	100	0	84.96
4	1670	147876222	C	Intron	NA	NA	NA	NA	NA	NA
4	1816	163181066	A	Intron	*Eleutherodactylus coqui*	KAG9479171.1 hypothetical protein GDO78_012704 [ *Eleutherodactylus coqui* ]	362	100	0	84.81
4	2866	258571195	A	Intergenic	*Ranitomeya imitator*	CAJ0935123.1 unnamed protein product [ *Ranitomeya imitator* ]	2581	95	0	61.19
4	2867	258571195	A	Intergenic	NA	NA	NA	NA	NA	NA
5	197	12384564	*Bd*	Intergenic	*Bufo gargarizans*	XP_044125285.1 uncharacterized protein C11orf96 homologue [ *Bufo gargarizans* ]	112	100	8.00E‐51	84.82
5	198	12384564	*Bd*	Intergenic	*Phyllobates terribilis*	XP_073515551.1 alpha‐ketoglutarate‐dependent dioxygenase alkB homologue 3 isoform X2 [ *Phyllobates terribilis* ]	278	100	4.00E‐179	85.25
5	288	16861628	C	CDS (synonymous)	*Bufotes viridis*	CAK8650277.1 Aryl hydrocarbon receptor nuclear translocator‐like protein 1 [ *Bufotes viridis* ]	223	100	1.00E‐140	95.07
5	680	40171501	*Bd*	Intergenic	*Xenopus laevis*	XP_041422148.1 uncharacterized protein LOC121394663 [ *Xenopus laevis* ]	964	43	6.00E‐51	33.12
5	681	40171501	*Bd*	Intergenic	NA	NA	NA	NA	NA	NA
5	1018	58881642	*Bd*	Intergenic	*Cimex lectularius*	XP_014253997.1 uncharacterized protein LOC106669195 [ *Cimex lectularius* ]	194	44	8.00E‐04	35.29
5	1019	58881642	*Bd*	Intergenic	NA	NA	NA	NA	NA	NA
5	1098	66373608	A	Intron	*Bufotes viridis*	CAK8649036.1 Rho family‐interacting cell polarization regulator 1 [ *Bufotes viridis* ]	409	49	2.00E‐117	89.6
5	1142	69807525	*Bd*	Intergenic	*Ranitomeya imitator*	CAJ0936902.1 unnamed protein product [ *Ranitomeya imitator* ]	142	46	1.00E‐13	69.57
5	1143	69807525	*Bd*	Intergenic	NA	NA	NA	NA	NA	NA
5	1148	70150765	*Bd*	Intron	*Bufo bufo*	XP_040272583.1 uncharacterized protein LOC120988885 [ *Bufo bufo* ]	276	62	3.00E‐55	50.57
5	1193	74908611	*Bd*	Intergenic	NA	NA	NA	NA	NA	NA
5	1194	74908611	*Bd*	Intergenic	*Hyla sarda*	XP_056376039.1 uncharacterized protein LOC130273369 isoform X2 [ *Hyla sarda* ]	547	35	2.00E‐18	34.72
5	1632	110638333	*Bd*	Intergenic	*Bufotes viridis*	CAK8648427.1 Cytochrome b‐c1 complex subunit Rieske mitochondrial partial [ *Bufotes viridis* ]	135	100	2.00E‐95	97.04
5	1633	110638333	*Bd*	Intergenic	NA	NA	NA	NA	NA	NA
5	2024	130513598	*Bd*	Intergenic	*Pseudophryne corroboree*	XP_063785579.1 uncharacterized protein LOC134933980 [ *Pseudophryne corroboree* ]	275	80	1.00E‐91	63.51
5	2025	130513598	*Bd*	Intergenic	NA	NA	NA	NA	NA	NA
5	2234	141581112	A	Intergenic	*Bufotes viridis*	CAK8638418.1 Speedy protein 1‐A [ *Bufotes viridis* ]	225	99	3.00E‐91	62.34
5	2235	141581112	A	Intergenic	*Eleutherodactylus coqui*	XP_066450754.1 CUB and zona pellucida‐like domain‐containing protein 1 [ *Eleutherodactylus coqui* ]	159	99	8.00E‐88	79.11
5	2417	158192647	A	Intron	Hylinae	XP_056380358.1 leucine‐rich repeat and transmembrane domain‐containing protein 1 isoform X3 [ *Hyla sarda* ]	318	100	0	89.27
5	2425	158989549	A	Intron	*Engystomops pustulosus*	KAG8556962.1 hypothetical protein GDO81_018267 [ *Engystomops pustulosus* ]	209	100	8.00E‐147	97.13
5	2635	173187959	C	Intergenic	*Penaeus vannamei*	XP_069975273.1 helicase SRCAP‐like [ *Penaeus vannamei* ]	696	92	2.00E‐13	35.88
5	2636	173187959	C	Intergenic	*Hyla sarda*	XP_056379887.1 Trio and F‐Actin‐binding protein isoform X1 [ *Hyla sarda* ]	1480	93	0	57.52
6	58	2609165	C	Intergenic	*Ephydatia muelleri*	KAL5466966.1 hypothetical protein EMCRGX_G031131 [Ephydatia muelleri]	182	93	7.00E‐27	40
6	59	2609165	C	Intergenic	*Bufotes viridis*	CAK8641417.1 hypothetical protein BUFVIR_LOCUS21661 partial [ *Bufotes viridis* ]	1243	62	0	64.04
6	870	41960955	C	Intergenic	Xenopus	XP_018085499.1 sodium/hydrogen exchanger 6 [ *Xenopus laevis* ]	395	99	0	85.71
6	871	41960955	C	Intergenic	Hyloidea	XP_066437927.1 sodium/hydrogen exchanger 6 [ *Eleutherodactylus coqui* ]	99	87	3.00E‐49	93.02
6	1144	67069274	A	Intron	Hyloidea	XP_056396842.1 mitochondrial import inner membrane translocase subunit Tim8 A isoform X3 [ *Hyla sarda* ]	87	100	5.00E‐52	90.8
6	1377	84763929	A	Intergenic	Dendrobatinae	XP_069603394.1 forkhead box protein O4 [ *Ranitomeya imitator* ]	484	100	0	91.19
6	1378	84763929	A	Intergenic	*Engystomops pustulosus*	XP_071982009.1 uncharacterized protein CXorf65 homologue [ *Engystomops pustulosus* ]	225	24	2.00E‐12	63.64
6	2359	139815076	A	Intergenic	*Bufotes viridis*	CAK8625270.1 TREH partial [ *Bufotes viridis* ]	463	84	0	73.24
6	2360	139815076	A	Intergenic	*Hyla sarda*	XP_056400770.1 nicotinamide N‐methyltransferase‐like isoform X1 [ *Hyla sarda* ]	484	65	4.00E‐172	80.88
6	2372	141400786	C	Intron	*Dendropsophus ebraccatus*	XP_069804661.1 out at first protein homologue isoform X1 [ *Dendropsophus ebraccatus* ]	310	79	1.00E‐155	81.23
6	2690	168489994	C	Upstream	*Ranitomeya imitator*	CAJ0934254.1 unnamed protein product [ *Ranitomeya imitator* ]	132	100	1.00E‐89	97.73
7	717	54320318	C	Intergenic	*Ranitomeya imitator*	CAJ0957900.1 unnamed protein product [ *Ranitomeya imitator* ]	405	100	0	96.54
7	718	54320318	C	Intergenic	Hylinae	XP_056411103.1 evC complex member EVC isoform X1 [ *Hyla sarda* ]	921	100	0	90.66
7	987	76048410	*Bd*	Intergenic	*Dendropsophus ebraccatus*	XP_069833732.1 vascular endothelial growth factor receptor 2 [ *Dendropsophus ebraccatus* ]	1287	100	0	81.89
7	988	76048410	*Bd*	Intergenic	NA	NA	NA	NA	NA	NA
7	1113	85582971	*Bd*	Intergenic	Neobatrachia	XP_066429596.1 deoxycytidylate deaminase [ *Eleutherodactylus coqui* ]	161	96	2.00E‐103	90.91
7	1114	85582971	*Bd*	Intergenic	Neobatrachia	XP_066429601.1 teneurin‐3 isoform X4 [ *Eleutherodactylus coqui* ]	1044	100	0	98.75
7	1162	91052400	*Bd*	Intergenic	*Eleutherodactylus coqui*	XP_066445357.1 uncharacterized protein [ *Eleutherodactylus coqui* ]	361	80	4.00E‐80	50.32
7	1163	91052400	*Bd*	Intergenic	*Pseudophryne corroboree*	XP_063807533.1 uncharacterized protein LOC134999973 [ *Pseudophryne corroboree* ]	519	71	9.00E‐104	43.32
7	1220	97819922	A	Intergenic	*Bufotes viridis*	CAK8601763.1 Retrovirus‐related Pol polyprotein from transposon 412 partial [ *Bufotes viridis* ]	376	100	5.00E‐97	49.85
7	1221	97819922	A	Intergenic	*Hyla sarda*	XP_056425863.1 uncharacterized protein LOC130367467 [ *Hyla sarda* ]	233	53	2.00E‐45	62.1
7	1297	105410216	*Bd*	Intergenic	Tetrapoda	NP_001362451.1 tripartite motif‐containing protein 2 isoform 16 [ *Homo sapiens* ]	267	100	0	97.75
7	1298	105410216	*Bd*	Intergenic	*Pyxicephalus adspersus*	XP_072262028.1 tripartite motif‐containing protein 2 [ *Pyxicephalus adspersus* ]	483	99	0	98.11
7	1397	114398105	C	Intergenic	*Ranitomeya imitator*	XP_069587485.1 uncharacterized protein [ *Ranitomeya imitator* ]	606	97	1.00E‐121	58.19
7	1398	114398105	C	Intergenic	*Pelobates cultripes*	CAH2321252.1 hypothetical protein XELAEV_18041981mg [ *Pelobates cultripes* ]	519	97	6.00E‐60	31.42
7	1512	123033970	*Bd*	Intergenic	NA	NA	NA	NA	NA	NA
7	1513	123033970	*Bd*	Intergenic	NA	NA	NA	NA	NA	NA
7	1519	123970626	C	Intergenic	*Pseudophryne corroboree*	XP_063781315.1 uncharacterized protein LOC134929662 [ *Pseudophryne corroboree* ]	519	29	3.00E‐25	43.67
7	1520	123970626	C	Intergenic	NA	NA	NA	NA	NA	NA
7	1669	139679428	*Bd*	Intergenic	*Brachypodium distachyon*	KQK19771.1 hypothetical protein BRADI_1g50390v3 [ *Brachypodium distachyon* ]	149	66	7.00E‐12	31.31
7	1670	139679428	*Bd*	Intergenic	*Bufo bufo*	XP_040274150.1 B‐cell scaffold protein with ankyrin repeats isoform X1 [ *Bufo bufo* ]	641	95	0	67.15
7	1698	142368845	*Bd*	Intergenic	*Hyla sarda*	XP_056422034.1 nephronectin isoform X2 [ *Hyla sarda* ]	381	99	4.00E‐169	70.41
7	1699	142368845	*Bd*	Intergenic	NA	NA	NA	NA	NA	NA
7	1805	150224597	*Bd*	Intron	NA	NA	NA	NA	NA	NA
7	2297	174138650	*Bd*	Intron	NA	NA	NA	NA	NA	NA
7	2311	174545091	C	Intergenic	NA	NA	NA	NA	NA	NA
7	2312	174545091	C	Intergenic	NA	NA	NA	NA	NA	NA
7	2405	178626031	A	Intergenic	NA	NA	NA	NA	NA	NA
7	2406	178626031	A	Intergenic	NA	NA	NA	NA	NA	NA
8	501	31946921	C	Intergenic	*Pseudophryne corroboree*	XP_063785579.1 uncharacterized protein LOC134933980 [ *Pseudophryne corroboree* ]	350	77	7.00E‐81	53.11
8	502	31946921	C	Intergenic	NA	NA	NA	NA	NA	NA
8	578	36784731	C	Intergenic	NA	NA	NA	NA	NA	NA
8	579	36784731	C	Intergenic	Bufonidae	CAK8660958.1 Urokinase‐type plasminogen activator [ *Bufotes viridis* ]	434	98	0	82.05
8	617	38371480	*Bd*	Intergenic	*Hyperolius riggenbachi*	XP_068111460.1 disintegrin and metalloproteinase domain‐containing protein 8 [ *Hyperolius riggenbachi* ]	941	74	0	69.92
8	618	38371480	*Bd*	Intergenic	NA	NA	NA	NA	NA	NA
8	746	46245647	C	Intron	Hyloidea	XP_044155964.1 cadherin‐related family member 1 [ *Bufo gargarizans* ]	368	94	0	95.65
8	759	47214853	C	Intergenic	*Dendropsophus ebraccatus*	XP_069836779.1 nuclear receptor coactivator 4 isoform X3 [ *Dendropsophus ebraccatus* ]	620	98	0	83.01
8	760	47493853	C	Intergenic	Anura	NP_001085741.1 macroH2A.2 histone S homeolog [ *Xenopus laevis* ]	371	100	0	93.82
8	760	47214853	C	Intergenic	Anura	NP_001085741.1 macroH2A.2 histone S homeolog [ *Xenopus laevis* ]	371	100	0	93.82
8	760	47417998	A	Intergenic	Anura	NP_001085741.1 macroH2A.2 histone S homeolog [ *Xenopus laevis* ]	371	100	0	93.82
8	761	47493853	C	Intergenic	Anura	XP_069835314.1 elastase‐1‐like [ *Dendropsophus ebraccatus* ]	240	90	4.00E‐152	95.83
8	761	47417998	A	Intergenic	Anura	XP_069835314.1 elastase‐1‐like [ *Dendropsophus ebraccatus* ]	240	90	4.00E‐152	95.83
8	835	51092147	C	Intron	NA	NA	NA	NA	NA	NA
8	835	50997880	C	Intron	NA	NA	NA	NA	NA	NA
8	1029	62903832	C	Intergenic	*Bufo gargarizans*	XP_044155649.1 alpha‐2‐macroglobulin‐like protein 1 [ *Bufo gargarizans* ]	749	98	0	73.37
8	1030	62903832	C	Intergenic	*Phyllobates terribilis*	XP_073541271.1 alpha‐2‐macroglobulin‐like protein 1 [ *Phyllobates terribilis* ]	642	100	0	68.64
8	1547	103103037	*Bd*	Intergenic	*Dallia pectoralis*	KAJ8013806.1 hypothetical protein DPEC_G00033610 [ *Dallia pectoralis* ]	1079	98	0	31.59
8	1548	103103037	*Bd*	Intergenic	NA	NA	NA	NA	NA	NA
8	1582	105358869	C	Intergenic	*Hyperolius riggenbachi*	XP_068098180.1 uncharacterized protein [ *Hyperolius riggenbachi* ]	152	100	4.00E‐98	92.76
8	1583	105358869	C	Intergenic	*Pseudophryne corroboree*	XP_063788599.1 uncharacterized protein LOC134943977 [ *Pseudophryne corroboree* ]	534	49	8.00E‐152	87.4
8	1902	129985695	A	Intergenic	Tetrapoda	XP_047918207.1 VWFA and cache domain‐containing protein 1 isoform X3 [ *Anser cygnoides* ]	914	99	0	88.16
8	1903	129985695	A	Intergenic	*Limosa lapponica baueri*	PKU30592.1 vwfa and cache domain‐containing protein 1 isoform x2 [ *Limosa lapponica baueri* ]	193	100	2.00E‐115	90.16
9	1094	63254941	*Bd*	Intergenic	*Bufotes viridis*	CAK8659612.1 V‐type proton ATPase 116 kDa subunit a1 partial [ *Bufotes viridis* ]	578	100	0	91.09
9	1095	63254941	*Bd*	Intergenic	NA	NA	NA	NA	NA	NA
9	1396	73207296	A	Intergenic	Anura	XP_063281614.1 protein LSM12 [ *Pelobates fuscus* ]	194	100	1.00E‐133	93.81
9	1397	73207296	A	Intergenic	*Pseudophryne corroboree*	XP_063819630.1 insulin‐like growth factor‐binding protein complex acid labile subunit [ *Pseudophryne corroboree* ]	353	97	0	90.67
9	1516	78649536	C	Intergenic	Anura	PIO39113.1 Mitogen‐activated protein kinase kinase kinase 3 [Aquarana catesbeiana]	616	100	0	93.69
9	1517	78649536	C	Intergenic	*Symbiodinium microadriaticum*	OLQ13442.1 Periaxin [ *Symbiodinium microadriaticum* ]	1260	99	4.00E‐60	45.26
10	837	45336775	C	Intron	Closterium sp. NIES‐68	GJP41619.1 hypothetical protein CLOM_g1267 [Closterium sp. NIES‐68]	627	58	1.00E‐43	33.42
10	863	46826550	C	Intergenic	*Bufotes viridis*	CAK8610461.1 LINE‐1 retrotransposable element ORF1 protein [ *Bufotes viridis* ]	554	39	2.00E‐36	38.29
10	864	46826550	C	Intergenic	*Bufotes viridis*	CAK8654144.1 LINE‐1 retrotransposable element ORF1 protein [ *Bufotes viridis* ]	477	64	8.00E‐72	44.34
10	876	47592404	C	Intergenic	*Dendrobatinae*	XP_069588333.1 mitochondrial ribonuclease P catalytic subunit [ *Ranitomeya imitator* ]	587	100	0	67.87
10	877	47592404	C	Intergenic	Anura	NP_001004923.1 serine/threonine‐protein phosphatase 2A regulatory subunit B″ subunit gamma [ *Xenopus tropicalis* ]	343	100	0	95.04
11	47	1452578	*Bd*	Intergenic	*Prochilodus magdalenae*	KAI4903493.1 hypothetical protein NFI96_006815 partial [ *Prochilodus magdalenae* ]	1250	29	1.00E‐13	26.67
11	48	1452578	*Bd*	Intergenic	NA	NA	NA	NA	NA	NA
11	602	17084627	A	Intron	Hyloidea	XP_056392891.1 parvalbumin beta‐like isoform X3 [ *Hyla sarda* ]	100	99	2.00E‐45	73.74
11	819	50470572	*Bd*	Intergenic	NA	NA	NA	NA	NA	NA
11	819	51648574	*Bd*	Intergenic	NA	NA	NA	NA	NA	NA
11	819	52230671	A	Intergenic	NA	NA	NA	NA	NA	NA
11	819	45982297	A	Intergenic	NA	NA	NA	NA	NA	NA

*Note:* For each candidate locus, the table lists the scaffold, gene identifier, SNP location, population comparison in which the locus was detected, and validated genomic context of the SNP (intergenic, intronic, CDS, upstream or downstream). The closest BLASTp match from the NCBI clusteredNR protein database is provided as the cluster ancestor and representative sequence, along with query length, query coverage percentage, E‐value and percentage sequence identity. Intergenic SNPs are shown with their nearest annotated flanking genes, whereas intronic and CDS SNPs occur within annotated gene spans. Entries marked NA indicate cases where no significant BLAST match was recovered.

Amino acid sequences from the corresponding genes were queried using BLASTp against the NCBI clusteredNR protein database, resulting in functional annotations for 167 unique protein sequences, while 48 sequences returned no significant matches (Table 3). Overlap among candidate loci was limited across analyses. At the gene level, scaffold 8 genes 760 and 761 were common to both the site A and site C temporal comparisons, while scaffold 11 gene 819 was shared between the *Bd* naïve versus endemic exposure contrast and the site A temporal comparison. Based on their biological roles, annotated genes were grouped into 11 functional categories: Immune‐related, Reproductive, Stress/Detoxification/Metabolism, Structural/Extracellular Matrix, Signalling/Transduction, Transport, Transcription/Translation, Cell Cycle/DNA Repair, Neurological/Behavioural, Retrotransposon/Transposase and Uncharacterized/Hypothetical (Figure 3).

**FIGURE 3:**
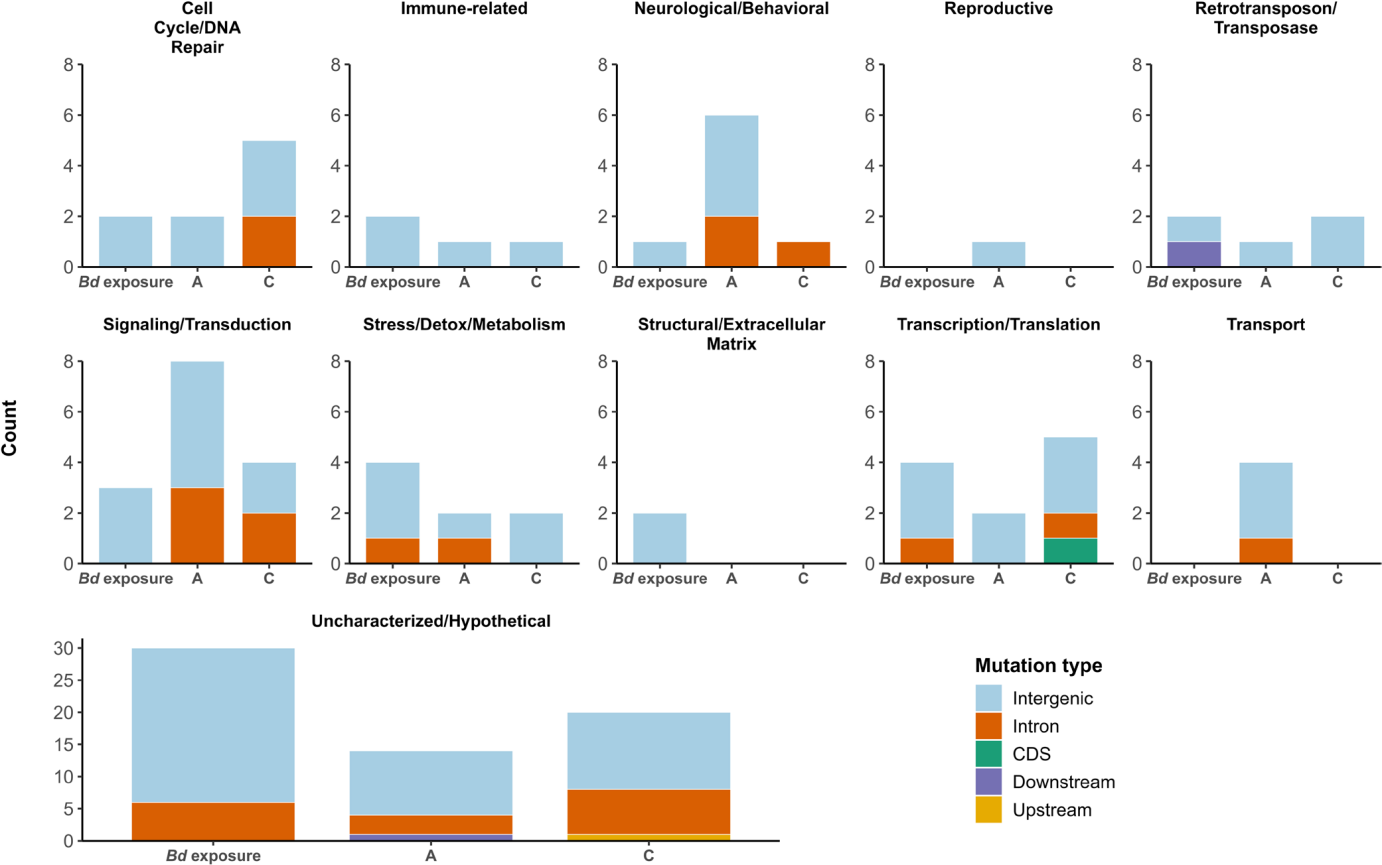
Stacked bar charts (faceted by functional category) display counts of annotated genes grouped by population sets *Bd*, A and C on the x‐axis; the y‐axis shows gene counts, with totals printed above each bar. Bar fills indicate genomic location from the legend (light blue: Intergenic, orange: Intron, green: CDS (coding sequence), purple: Downstream, yellow: Upstream). Functional categories shown include Cell Cycle/DNA Repair, Immune‐related, Neurological/Behavioural, Reproductive, Retrotranposon/Transposons, Signaling/Transduction, Stress/Detox/Metabolism, Structural/Extracellular Matrix, Transcription/Translation, Transport, and Unknown/Hypothetical.

The largest category across all analyses was Uncharacterized/Hypothetical, comprising 30 genes in the *Bd* naïve versus endemic exposure contrast, 14 in site A, and 20 in site C. Among characterized genes, Signalling/Transduction was the most represented functional category, particularly in the site A comparison, where 8 genes were assigned to this category, compared with 3 in the *Bd* naïve versus endemic exposure contrast and 4 in the site C comparison. Other categories with relatively high representation included Transcription/Translation, Stress/Detoxification/Metabolism, Neurological/Behavioural and Cell Cycle/DNA Repair. Immune‐related annotations were present but relatively uncommon, with 2 genes in the *Bd* naïve versus endemic exposure contrast comparison and one gene each in the site A and C comparisons. Reproductive and Transport‐related annotations were more restricted, occurring primarily in the site A comparison. Overall, 48% of genes were successfully assigned to functional categories, while 52% were labelled as Uncharacterized/Hypothetical. Full annotation details for candidate loci are provided in Data S1.

### 3.4 Comparison With Previous Bd‐Associated Transcriptomic Responses

To assess whether candidate‐associated genes identified in this study overlapped with previously reported *Bd*‐associated transcriptomic responses in *L. v. alpina* we compared our candidate annotations with the liver, skin and spleen transcriptome annotations from Grogan, Mulvenna, et al. (2018). Of the 225 SNP‐gene association records, 64 matched Grogan et al. annotations by exact accession identifier or standardized protein description (Data S1). These matches included immune‐related or immune‐adjacent genes such as toll‐like receptor 8, CD276 antigen, fibroleukin‐like and zinc finger and BTB domain‐containing proteins. Matches also included genes associated with cellular regulation, signalling, transcriptional regulation and reproduction‐related processes.

## 4 Discussion

Comparisons between *Bd*‐naïve and *Bd*‐endemic populations showed candidate loci predominantly in non‐coding regions, including intronic SNPs within annotated gene spans and intergenic regions, with limited direct evidence of immune‐gene involvement. Only ~2% of candidate loci were annotated as immune‐related, while the majority fell into transcriptional, signalling or uncharacterized categories. The scarcity of clear immune hits is notable, but not necessarily surprising. In *Litoria verreauxii alpina*, protective immunity to *Bd* appears limited (Grogan et al. 2023; Grogan, Skerratt, et al. 2018), which might reduce the scope for strong selection on classical immune resistance. In addition, because this species can recruit successfully despite high adult mortality, many susceptible individuals may survive long enough to reproduce before infection‐associated mortality removes them from the population. This life‐history pattern may weaken selection for classical immune resistance relative to systems where disease prevents many individuals from achieving reproductive success.

The comparison with Grogan, Mulvenna, et al. (2018) provides complementary support for interpreting candidate‐associated genes in the context of known *Bd*‐associated host responses in *L. v. alpina*. We identified 64 candidate‐associated genes which matched Grogan et al. transcriptome annotations by accession or standardized protein description. This overlap included immune‐related or immune‐adjacent genes, such as toll‐like receptor 8, CD276 antigen‐like and fibroleukin‐like, as well as genes associated with transcriptional regulation, signalling, cellular processes and reproduction‐related functions. These matches indicate that some differentiated genomic regions identified here are linked to biological processes previously observed during *Bd* infection in this species. Rather than providing a one‐to‐one validation of candidate loci, this comparison helps prioritize genes and functional categories for future work that can directly test links among genotype, gene expression, infection load, survival and reproduction.

The relatively recent timeframe of *Bd* exposure in alpine environments (Fisher et al. 2009; Scheele et al. 2017), together with the prevalence of non‐coding variants in adaptive differentiation (Ellingford et al. 2022; Wu et al. 2024), suggests that strong immune adaptation may not be the most likely outcome in this system. Instead, the concentration of candidates in signalling and transcriptional control suggests that pathogen‐associated genomic responses in this system may be distributed across upstream regulatory networks rather than centred on a small set of canonical immune loci, a pattern broadly consistent with polygenic models of adaptation (Barghi et al. 2020; Quintana‐Murci 2019). A recent genomic study of recovering *Rana sierrae* also found that candidate loci were spread across genes involved in skin integrity, intracellular trafficking, metabolic stress and interferon‐related processes, with no evidence that recovery was driven by a single enriched functional pathway (Byrne et al. 2026). This pattern also increases the value of the present candidate list as a resource for future work, because it highlights a tractable set of loci and biological processes that can now be tested more directly in *L. v. alpina* and compared with *Bd*‐associated candidates in other amphibian systems.

### 4.1 Temporal Shifts Within Populations

Temporal contrasts revealed population‐specific differences in candidate loci. In site C, selection signals were diffuse and dominated by signalling and cell‐cycle pathways, with minimal evidence of immune‐related candidates. Differences in effective population size may help explain some of the contrast between sites, because populations with larger effective sizes are generally expected to experience a weaker influence of drift and a greater efficacy of selection (Charlesworth 2009; Nielsen 2005). In that context, the pattern at site C provides little support for a strong genomic response centered on classical immune loci over the timeframe examined here, which is consistent with the broader view that *L. v. alpina* may have limited capacity for protective immune adaptation to *Bd*.

In contrast, site A showed clear evidence of reproduction‐related candidate loci (testis development‐related protein), alongside signalling and neurobehavioral categories. This is noteworthy because reproductive compensation is one of the recognized mechanisms by which frog populations persist with *Bd* (Brannelly, Webb, et al. 2016; Brannelly, Webb, et al. 2021; Lee et al. 2025; Valenzuela‐Sánchez et al. 2022). However, the presence of a reproduction‐related candidate should not be interpreted as evidence that selection is stronger at site A. If site A has a smaller effective population size, drift may play a stronger role there, increasing the chance that alleles shift stochastically even in the absence of strong selection (Charlesworth 2009; Nielsen 2005). Rather, the pattern at site A may indicate that any pathogen‐associated genomic change occurring in this population is not centred on classical immune resistance. Instead, the candidate loci span several functional categories, including signalling/transduction, neurological/behavioural, transport and reproductive biology. Although a reproduction‐related candidate was identified, the candidate loci at site A were distributed across several functional categories. Taken together, these contrasting patterns suggest that demographic differences among populations, including possible differences in effective population size, may influence how genomic responses to *Bd* are expressed across sites, even if those demographic effects cannot be disentangled directly here.

Importantly, overlap among candidate loci across the *Bd*‐naïve versus endemic exposure contrast, site A temporal comparison, and site C temporal comparison was limited. Given that all three comparisons might capture some aspect of response to *Bd*, greater overlap might have been expected if similar selective pressures were repeatedly acting on a shared set of loci, although incomplete parallelism is common even under similar environments (Bolnick et al. 2018; Hartke et al. 2021). Instead, the low overlap suggests that any response to *Bd* may be heterogeneous, context dependent, distributed across different pathways or partly obscured by drift and incomplete genomic resources or annotation (Bolnick et al. 2018; Formenti et al. 2022; Hartke et al. 2021; Hoban et al. 2016). Because SNP filtering was performed separately for each contrast, differences in SNP number and marker composition may also reduce apparent overlap among candidate sets. For this reason, overlap was interpreted descriptively rather than as a formal test of parallel selection. It is also possible that many of these changes reflect a *Bd*‐driven shift in life history, whereby mass mortality prevents most individuals from reaching older age classes, leaving predominantly young animals to reproduce and altering the selection pressures experienced by the population. Limited connectivity among populations may also contribute to this pattern by restricting the spread of beneficial variants and allowing more independent evolutionary trajectories among populations through time (Morjan and Rieseberg 2004; Tigano and Friesen 2016). These candidate loci should also be interpreted in the context of demographic processes that can shape allele‐frequency change in remnant populations. Drift, bottlenecks, restricted connectivity, age‐structured mortality and repeated recruitment of young breeders following adult mortality might all influence patterns of genomic differentiation across sites or through time (Charlesworth 2009; Heard et al. 2024; Holsinger and Weir 2009; Lowe and Allendorf 2010; Scheele et al. 2016). In *L. v. alpina*, this is especially relevant because *Bd*‐associated mortality can be severe and population persistence appears to depend heavily on recruitment and compensatory reproductive effort (Brannelly et al. 2025; Brannelly, Webb, et al. 2021; Scheele et al. 2015). Thus, elevated differentiation might reflect pathogen‐associated selection, demographic change associated with disease‐driven turnover or a combination of both. As in the *Bd*‐naïve versus endemic exposure contrast, these loci should be interpreted primarily as gene‐associated, non‐coding variants rather than confirmed protein‐altering substitutions, because non‐coding regulatory variation can make important contributions to adaptive phenotypic change (Wray 2007).

### 4.2 Broader Evolutionary Context and Limitations

The results underscore that disease can shape host genomes over relatively short timescales, because adaptive responses can emerge over tens of generations and may involve either large allele‐frequency shifts at a few loci or more modest shifts across many loci (Cassin‐Sackett et al. 2025; Stephan 2021). Although most detected SNPs here were intergenic, many loci were linked to uncharacterized or hypothetical genes, highlighting the continuing limitations of amphibian genome annotation and reference resources (Kosch et al. 2024, 2025). In alpine tree frogs, *Bd* is thought to have arrived in the early 1980s, and the exposed populations used in experimental work had experienced *Bd* for approximately 26 years (Grogan, Mulvenna, et al. 2018). Over that interval, however, this species has remained highly susceptible to *Bd*, with low recovery from infection and near‐complete annual turnover in endemic remnant populations, suggesting that adaptive responses may be constrained by severe ongoing disease impacts rather than progressing rapidly toward robust resistance or population resilience (Brannelly et al. 2015; Grogan, Mulvenna, et al. 2018). More broadly, *Bd* can suppress amphibian lymphocyte responses, and protective acquired immunity appears to be uncommon across amphibians, which supports caution in assuming that strong adaptation is inevitable (Carlson et al. 2014; Fites et al. 2013; Grogan et al. 2023). Even so, modest allele‐frequency shifts may still be consistent with the early stages of longer‐term polygenic change rather than the absence of selection altogether (Stephan 2021).

Pathogen‐driven selection in other taxa shows how initially subtle signals can, over time, translate into broader evolutionary responses. In Tasmanian devils, exposure to the transmissible facial tumour disease has produced rapid allele frequency shifts not only in immune‐related genes but also in pathways linked to cell adhesion and apoptosis, reflecting secondary changes that may improve tumour recognition and tissue compatibility (Epstein et al. 2016; Margres et al. 2018). Similarly, following the introduction of myxoma virus in European rabbits, early mortality was catastrophic, yet subsequent generations exhibited selection across multiple immune signalling and inflammatory pathways rather than a single locus, highlighting the polygenic and pathway‐level nature of long‐term adaptation to novel pathogens (Alves et al. 2019; Kerr et al. 2015).

Together, these cases demonstrate that disease selection rarely acts in isolation on a single gene, but instead can reshape interacting pathways, with small and diffuse shifts gradually accumulating into larger evolutionary outcomes, a pattern consistent with modern views of polygenic adaptation (Barghi et al. 2020; Hayward and Sella 2022; Höllinger et al. 2019; Jain and Stephan 2017). However, they also highlight that such outcomes depend on the pathogen imposing strong and consistent fitness differentials across generations. In alpine frogs, the present results suggest that *Bd* causes high adult mortality, but many individuals might still reproduce before dying (Brannelly, Webb, et al. 2021; Crawford‐Ash et al. 2024; Scheele et al. 2015). This life‐history pattern could reduce the strength of selection for classical resistance while increasing the importance of recruitment, compensatory reproduction and demographic turnover. In that context, the scattered allele frequency changes observed here may represent either early adaptive trajectories to mass mortality due to disease or the genomic signature of persistence under constrained adaptive potential. They might also reflect demographic responses to altered life history, or a combination of selection and demographic change, which could help explain the variability of genomic outcomes across affected populations.

A key limitation of the present study is the reliance on SNP‐level signals without functional validation, particularly since most candidate loci are intergenic. Noncoding variants can contribute to adaptation through effects on gene regulation, but their functional consequences cannot be inferred from genomic position alone (Findley et al. 2021; Wray 2007). In addition, validation against the reference GFF3 indicated that many variants initially parsed as occurring in genes were intronic, reinforcing that most candidate loci identified here are non‐coding and should not be assumed to alter protein sequences directly. BayPass identifies loci with elevated differentiation while accounting for covariance among groups, but elevated differentiation can also reflect demographic processes, including drift, bottlenecks, age‐structured mortality or population‐specific allele‐frequency change (Gautier 2015; Lotterhos and Whitlock 2014). Future work should incorporate approaches that directly link these variants to gene regulation in tissues relevant to chytrid infection.

One particularly useful next step would be to assess these candidate regions against experimental infection datasets in more detail. Previous work with *Litoria verreauxii alpina* has already examined gene expression and metabolite responses under *Bd* infection, revealing distinct transcriptional and physiological shifts associated with survival outcomes (Grogan, Mulvenna, et al. 2018). Our descriptive comparison with Grogan, Mulvenna, et al. (2018) provides an initial step in this direction by identifying candidate‐associated genes that correspond to previously reported *Bd*‐associated transcriptomic annotations. Pairing the candidate SNPs identified here with experimentally infected individuals would allow a more direct test of whether the loci highlighted correspond to expression changes, physiological responses, infection load or survival under controlled challenge. More broadly, work on context‐dependent gene regulation has shown that many noncoding regulatory effects become most apparent under specific cellular or environmental conditions, including immune activation (Findley et al. 2021; Soskic et al. 2022). Likewise, infection‐focused single‐cell and single‐nucleus transcriptomic studies in other taxa, including silkworm midgut tissue following nucleopolyhedrovirus infection, demonstrate how pathogen challenge can reveal cell‐type specific immune responses that are otherwise difficult to detect (Xia et al. 2024). Together, these approaches illustrate how the candidate loci identified here could be tested more directly against experimentally measured *Bd* responses.

### 4.3 Comparative Value and Future Directions

This study contributes to a growing body of work on evolution with endemic wildlife disease, demonstrating that even within a single species, genomic responses may differ depending on demography, exposure history and local population size. The contrasting trajectories at sites A and site C emphasize that evolutionary outcomes are context‐dependent across populations, and that persistence does not necessarily imply the evolution of strong immune resistance.

Importantly, the candidate loci identified here provide more than a set of outliers from a single focal system. They represent a practical starting point for future work on *Bd*‐associated genomic responses, including functional validation within *L. v. alpina*, comparison with transcriptomic and infection datasets, and cross‐species tests of whether similar regulatory, signalling or reproduction‐related pathways recur in other amphibians exposed to *Bd*. A portion of this study was made possible by archival tissue banks, which enabled direct temporal comparisons and highlight the broader value of preserved samples and temporal genomics for studying contemporary evolution in wildlife (Benham and Bowie 2023; Clark et al. 2023; Jensen and Leigh 2022). More broadly, the expanding amphibian genomics toolkit should make it increasingly feasible to test whether the predominantly noncoding and weakly shared signatures identified here recur across other *Bd*‐affected taxa, increasing the comparative value of these candidate loci beyond this focal species (Kosch et al. 2024). Looking forward, integrating genomic scans with experimental infections, expression data and demographic monitoring will be essential for determining whether these candidate regions are linked to infection load, survival, tolerance and compensatory reproduction instead reflect altered life history, demographic change or a combination of these processes. In this sense, the present study provides both biological inference about pathogen‐associated genomic change and a comparative resource for future amphibian disease genomics.

## Author Contributions

A.S.W. and L.A.B. conceived and designed the study; A.S.W. and L.A.B. collected data; A.S.W. analysed data; A.S.W. and L.A.B. drafted and edited the manuscript; A.S.W., L.B., L.F.S. and L.A.B. provided intellectual input and edited the manuscript.

## Funding

The study was funded by the American Australian Association; the Holsworth Wildlife Research Endowment—Equity Trustees Charitable Foundation, the Ecological Society of Australia; the Australian Research Council (DE180101395); the Australian Academy of Science (Max Day Environmental Science Fellowship); Bioplatforms Australia as part of the Threatened Species Initiative; and the Dr. Albert Shimmins Fund.

## Disclosure

Benefits from this research accrue through the public sharing of raw genomic data, sample metadata and annotated candidate loci as described above. This study also contributes to the growing understanding of genes and genomic regions potentially influenced by *Batrachochytrium dendrobatidis* in wild amphibian populations by identifying candidate loci, functional annotations and pathways that can be used in future comparative and functional studies within this species and across other *Bd*‐affected amphibians. More broadly, the research addresses a priority conservation concern by improving understanding of the genomic basis of persistence under endemic wildlife disease and by providing resources that may support future research and management.

## Ethics Statement

All work was conducted under approval of the University of Melbourne's Animal Ethics Committee (application 1814537), the Wildlife Act 1975 Research Authorization permit numbers 10008829, and the NSW scientific licence SL102890 issued under the Biodiversity Conservation Act 2016.

## Conflicts of Interest

The authors declare no conflicts of interest.

## Supporting information


**Data S1:** Functional annotation of candidate SNP‐associated loci identified from selection scans in *Litoria verreauxii alpina*. The table contains 225 annotation records for candidate loci from the *Bd* exposure contrast and temporal comparisons at Site A and Site C. Columns include scaffold and gene identifiers, SNP position and genomic classification, BLAST query and match statistics, best‐matching accession and taxonomic/common‐name information, assigned functional description and functional grouping, and overlap with liver, skin and spleen transcriptome records from Grogan, Mulvenna, et al. (2018). NA indicates that no annotation or transcriptome match was identified for that field.

## Data Availability

Raw DArTseq data, associated sample metadata, and R code for genomic analyses are openly available in Figshare at [https://doi.org/10.26188/32148115]. The transcriptome FASTA file used for annotation is openly available in Figshare at [https://doi.org/10.26188/28823072], as reported in Wendt and Brannelly et al. (2025).
